# *Lactobacillus paracasei* L9 affects disease progression in experimental autoimmune neuritis by regulating intestinal flora structure and arginine metabolism

**DOI:** 10.1186/s12974-023-02808-8

**Published:** 2023-05-22

**Authors:** Yuting Meng, Xiangjie Qiu, Zhongxiang Tang, Yu Mao, Yurong Tan

**Affiliations:** Department of Medical Microbiology, Xiangya School of Medicine, Central South University, Changsha, 410078 Hunan China

**Keywords:** Experimental autoimmune neuritis, *Lactobacillus paracasei* L9, Arginine and proline metabolism, Therapy

## Abstract

**Background:**

Autoimmune neuropathies are common peripheral nervous system (PNS) disorders. Environmental influences and dietary components are known to affect the course of autoimmune diseases. Intestinal microorganisms can be dynamically regulated through diet, and this study combines intestinal microorganisms with diseases to open up new therapeutic ideas.

**Methods:**

In Lewis rats, a model of EAN was established with P0 peptide, *Lactobacillus* were used as treatment, serum T-cell ratio, inflammatory factors, sciatic neuropathological changes, and pathological inflammatory effects on intestinal mucosa were detected, and fecal metabolomics and 16 s microbiome analysis were performed to further explore the mechanism.

**Results:**

In the EAN rat model, *Lactobacillus paracasei* L9 (LP) could dynamically regulate the CD4^+^/CD8^+^T balance in serum, reduce serum IL-1, IL-6 and TNF-α expression levels, improve sciatic nerve demyelination and inflammatory infiltration, and reduce nervous system score. In the rat model of EAN, intestinal mucosa was damaged. Occludin and ZO-1 were downregulated. IL-1, TNF-α and Reg3γ were upregulated. LP gavage induced intestinal mucosa recovery; occludin and ZO-1 upregulation; IL-1, TNF-α and Reg3γ downregulation. Finally, metabolomics and 16 s microbiome analysis were performed, and differential metabolites were enriched with an important metabolic pathway, arginine and proline metabolism.

**Conclusion:**

LP improved EAN in rats by influencing intestinal community and the lysine and proline metabolism.

## Background

Guillain–Barré syndrome (GBS) is an autoimmune disease of the peripheral nervous system (PNS) that is clinically characterized by acute flaccid paralysis and/or sensory/autonomous nerve dysfunction [1]. The annual incidence of GBS is 0.81–1.89 per 100,000 people worldwide, and appears to be increasing exponentially, along with increasing age in Western countries [2]. Currently, intravenous administration of immunoglobulin or plasma exchange is the optimal treatment approach [3, 4]. However, 3–10% of patients with GBS do not survive, while 20% live with severe disabilities [5]. The two most common forms of GBS are acute inflammatory demyelinating polyradiculoneuropathy (AIDP) and acute motor axonal neuropathy (AMAN) [6]. Experimental autoimmune neuritis (EAN) is the classical animal model for GBS and mimics the pathological and immunological features of GBS. Similar to GBS, EAN is characterized by activated T cells and macrophage infiltration into the PNS, inflammatory demyelination, and axonal injury of peripheral nerves [5, 7].

In recent years, people have called the intestinal flora the 8th organ of the human body, revealing the close connection between microorganisms and the human body, involved in mucosal protection, immune regulation, metabolic activity, etc. Phagocytosis and bactericidal activity of macrophages against various pathogens, such as *S. aureus*, *S. typhimurium*, and *E. coli*, were increased by pretreatment with *Lactobacillus.* Taken together, the experiments show that probiotic strains of *Lactobacillus* exert early immunostimulatory effects that may be directly related to the initial inflammation of the response of human macrophages [8]. In particular, *Lactobacilli* has been shown to be associated with autoimmune diseases [9]. For example, *L. delbrueckii* and *L. rhamnosus* have been shown to be effective in increasing Regulatory T cells (Tregs) and decreasing inflammatory cytokines and disease severity in mice induced by systemic lupus erythematosus [10]. Mu et al. found that *Lactobacillus* supplementation plays an anti-inflammatory role by reducing IL-6 and enhancing IL-10 generation in the gut [11, 12]. *Lactobacilli* can produce cytoprotective compounds, including short-chain fatty acids (SCFAs), which act as local energy carriers for bacteria and gut epithelial cells, have emerged as key mediators in the relationship between diet, gut microbiota, and human health [13, 14]. SFCAs could stimulate retinoic acid generation by epithelial cells [15], a vitamin A-derived metabolite that cooperates with TGF-β to enhance regulatory T cells (Tregs) differentiation [16] and prevent Th17 cell differentiation. Tregs and helper T cells (Th) play an important role in controlling immune responses and induction of peripheral tolerance. Many species of *Lactobacillus* have been reported to reprogram CD4^+^T cells into immunoregulatory T cells as well as regulating Th cells and Tregs [17].

At present, there is no effective treatment for EAN, and the onset of EAN is closely related to immune regulation of T cells and inflammatory infiltration of peripheral nerves, which can predict that *Lactobacillus* is a potential treatment for EAN. Through some research reports, *L. paracasei L9* and *L. bulgaricus* can be found to be closely related to the body’s immune regulation and inflammatory response, but less associated with the study of EAN. *L. paracasei* R3 protects against dextran sulfate sodium (DSS)-induced colitis in mice by regulating Th17/Treg cell balance [18, 19]. Consumption of Dairy Yogurt containing *L. paracasei* ssp. paracasei, *Bifidobacterium animalis* ssp. lactis and heat-treated *L. plantarum* improve immune function including natural killer cell activity [19]. *L. bulgaricus* and *L. plantarum* improve diabetic wound healing by modulating inflammatory factors [20]. Based on these previous studies, we believe lactobacillus can be used as a new strategy for EAN.

## Materials and methods

### Animals, in vivo treatment with Lactobacillus and experimental design

A total of 6- to 8-week-old female Lewis rats (n = 24) were purchased from Beijing Vital River Laboratory Animal Technology Co, and kept under standard conditions in our local animal facility (Laboratory Animal Management Center, Central South University) in pathogen-free cages with food and water available ad libitum. Animals were in the 140–160 g weight range on arrival and could acclimatize for at least 1 week. At experimental onset, animals were repeatedly weighed and randomly divided into four groups, the control group, the EAN model group, and the experimental groups (*n* = 6). The experimental groups were continuously treated with *L. bulgaricus* (CGMCC no.6970) or *L. Paracasei* L9 (CGMCC no. 9800) for 18 days from 1 week before the experiment to animal killing, and the control group and the model group were treated with normal saline. According to the literature,* L. bulgaricus*, and *L. paracase*i needed to be cultured at 37 °C aerobic environment and the bacterial concentration of gavage was 5 × 10^9^.

### Induction of EAN and evaluation of clinical score

In the therapeutic setting, EAN was induced with neurogenic P0 peptide (300 μg) (MCE, New Jersey, USA), which was emulsified in complete Freund adjuvants containing 1 mg/mL *Mycobacterium tuberculosis* H37RA (MCE, New Jersey, USA) and injected s.c. into the tail base. Booster after seven days. Disease onset was assessed daily by a blinded investigator using the following EAN score system: 0 normal; 1 impaired righting/limb tail; 2 absent righting; 3 ataxic gait/abnormal position; 4 mild paraparesis; 5 moderate paraparesis; 6 severe paraplegias; 7 tetra paresis; 8 moribund; 9 deaths.

### Flow cytometric analysis of mononuclear cells in blood

At the beginning of the disease (11 days) and at the peak of the disease (19 days), 200 μL of rat tail vein whole blood, three volumes of red blood cell lysate (Soleborg, Beijing, China) are lysed twice to obtain lymphocytes, and CD3^+^T CD4^+^T CD8^+^T (Biolegend, California, USA) surface staining is performed according to instructions.

### Histopathological analysis of sciatic nerve

Dissected sciatic nerves were divided into four sections of equal length and cryosections (8 μm) were prepared. Cryosections were observed for morphological and structural changes and axonal demyelination. The proximal spinal cord segment of the sciatic nerve was stained for HE staining to assess the degree of inflammatory cell infiltration. Each specimen randomly took 5 fields of view for inflammatory cell counts. The sciatic nerve is stained with luxol fast blue (LFB) to observe nerve demyelination. Colon specimens were stained for HE and Alixinlan staining to assess the degree of inflammatory infiltration of the colic and the integrity of the mucous membrane.

### Quantitative PCR

Dissected sciatic nerves and gut were immediately flash-frozen in liquid nitrogen and stored at − 80 °C. Frozen samples were lysed and homogenized with TRIzol™ reagent (Soleborg, Beijing, China), using the Tissue Lyser II system and Grinding beads (Servicebio, Wuhan, China). Using Reverse Transcription Mix Oligo (dT) and random primers (Takara, Beijing, China) to transcribe into cDNA according to the manufacturer’s protocol. Target-specific primers (Tsingke, Beijing, China) were used to evaluate relative mRNA expression levels of TNF-α, IL-1, occludin, Zo-1, Reg3γ, and qPCR were performed at 95℃ for denaturation, 60℃ for annealing/extension, and 40 cycles. Post-amplification melt-curve analysis was performed to verify primer–dimer arteficts and to verify reaction specificity. The relative target expression was determined using Pfaffl29 modified relative quantification model with efficiency correction and normalized to GAPDH and β-Actin as reference genes. Relative target gene expression is described as fold-change relative to the corresponding experimental control (CTR) group. All experiments were performed in triplicates, and mean Ct was used in the equation.

### Enzyme-linked immunosorbent reaction

Collect tail vein blood at the beginning (11 days) and peak (19 days), select EDTA as anticoagulant, mix for 10–20 min, centrifuge at 2–8 °C for about 20 min (2000–3000 rpm), and carefully collect supernatant. IL-1, IL-6 and TNF-α ELISA kits were purchased from Cusabio (Wuhan, China). Standards or samples (100 μL/well) were added and incubated for 2 h at room temperature. The liquid from the wells was then discard, the plate was washed three times with 1 × wash buffer (300 μL/well), and then the pre-formulated antibody was added (100 μL/well) and incubated at room temperature for 1 h. After washing, a color developer was added, and the absorbance at 450 nm was measured after stopping color development.

### Metabolomic analysis

Rat droppings were quickly frozen in liquid nitrogen immediately after dissection. The rat droppings were then cut on dry ice (~ 80 mg) into an Eppendorf tube (2 mL). Rat droppings with 200 μL of H_2_O and five ceramic beads were homogenized using the homogenizer. 800 μL of methanol/acetonitrile (1:1, v/v) were added to a homogenized solution for metabolite extraction. The mixture was centrifuged for 20 min (14,000 g, 4 °C). The supernatant was dried in a vacuum centrifuge. For LC–MS analysis, samples were resuspended in 100 μL acetonitrile/water (1:1, v/v) solvent and centrifuged at 14,000 g at 4 °C for 15 min, followed by supernatant injection. UHPLC-Q-TOF MS analysis was performed using UHPLC (1290 Infinity LC, Agilent Technologies) coupled to a quadrupole time-of-flight (AB Sciex TripleTOF 6600) in Shanghai Applied Protein Technology Co., Ltd. For hydrophilic action chromatography (HILIC) separation, samples were analyzed using a column 2.1 mm × 100 mm ACQUIY UPLC BEH Amide 1.7 µm (waters, Ireland). In both ESI positive and negative modes, the mobile phase contained A = 25 mM ammonium acetate and 25 mM ammonium hydroxide in water and B = acetonitrile. The gradient was 95% B for 0.5 min and was linearly reduced to 65% in 6.5 min, then reduced to 40% in 1 min and held for 1 min, then increased to 95% in 0.1 min, with a 3-min rebalancing period employed. The ESI source conditions were set as follows: Ion Source Gas1 (Gas1) as 60, Ion Source Gas2 (Gas2) as 60, Curtain Gas (CUR) as 30, source temperature: 600℃, Ion Spray Voltage Floating (ISVF) ± 5500 V. In MS acquisition only, the instrument was set to acquire over the m/z range 60–1000 Da, and the accumulation time for TOF MS scanning was set at 0.20 s/spectra. In auto MS/MS acquisition, the instrument was set to acquire over the m/z range 25–1000 Da, and the accumulation time for product ion scan was set at 0.05 s/spectra. The product ion scan is obtained using Information Dependent Acquisition (IDA) with a selected high sensitivity mode. The parameters were set as follows: collision energy (CE) was fixed at 35 V with ± 15 eV; declustering potential (DP), 60 V (+) and − 60 V (−); exclusion of isotopes within 4 Da, candidate ions to monitor per cycle: 10.

### Metabolomic data analysis

After sum-normalization, the processed data were analyzed by R package (ropls), where it was subjected to multivariate data analysis, including Pareto-scaled principal component analysis (PCA) and orthogonal partial least squares discriminant analysis (OPLS-DA). The sevenfold cross-validation and response permutation test was used to evaluate the robustness of the model. The variable importance in the projection (VIP) value of each variable in the OPLS-DA model was calculated to indicate its contribution to classification. Student’s *t* test was used to determine the significance of differences between two groups of independent samples. VIP > 1 and *p* < 0.05 were used to screen significant altered metabolites. Pearson’s correlation analysis was performed to determine the correlation between the two variables.

### 16S sequencing

Total genomic DNA from samples was extracted using the CTAB/SDS method. DNA concentration and purity were monitored on 1% agarose gels. Depending on the concentration, DNA was diluted to 1 ng/μL using sterile water. Amplicon Generation Primer: 16S V3-V4: 341F-806R, 18S V9: 1380F-1510R, ITS1: ITS1F- ITS2R. 16S/18S rRNA genes were amplified using the specific primer with the barcode. All PCR reactions were performed at 30 μL with 15 μL of Phusion High-Fidelity PCR Master Mix (New England Biolabs); 0.2 μM of forward and reverse primers, and approximately 10 ng of template DNA. Thermal cycling consisted of initial denaturation at 98 °C for 1 min, followed by 30 cycles of denaturation at 98 °C for 10 s, annealing at 50 °C for 30 s, and elongation at 72 °C for 60 s. Finally, 72 °C for 5 min. PCR products quantification was performed using electrophoresis on 2% agarose gel. Samples with bright main strips between 400 and 450 bp were selected for further experiments. PCR Products Mixing and purified PCR products were mixed in equal volume. Then, the PCR mixture was purified with an AxyPrepDNA Gel Extraction Kit (AXYGEN, Silicon Valley, USA). Library preparation and sequencing libraries were generated using NEB Next^®^Ultra™DNA Library Prep Kit for Illumina (NEB, USA) following manufacturer recommendations and index codes were added. The quality of library was assessed using the Qubit@2.0 Fluorometer (Thermo Scientific, Massachusetts, USA) and Agilent Bioanalyzer 2100 system. Finally, the library was sequenced on an Illumina NovaSeq 600 platform and 250 bp paired-end reads were generated.

### 16S data analysis

Paired-end reads assemblies Paired-end reads from the original DNA fragments were merged using FLASH, a very fast and accurate analysis tool that was designed to merge paired-end reads when at least some of the reads overlap the read generated from the opposite end of the same DNA fragment. Paired-end reads were assigned to each sample according to the unique barcodes. OTU cluster, species annotation and sequence analysis were performed by UPARSE software package using UPARSE-OTU and UPARSE-OTUref algorithms. In-house Perl scripts were used to analyze alpha (within samples) and beta (among samples) diversity. Sequences with ≥ 97% similarity were assigned to the same OTUs. We selected a representative sequence for each OTU and used the RDP classifier to annotate taxonomic information for each representative sequence. To compute Alpha Diversity, we verified the OTU table and calculated three metrics: Chao1 estimates the species abundance; Observed Species estimates the amount of unique OTUs found in each sample, and Shannon Index. Rarefaction curves were generated based on these three metrics. Phylogenic distance and community distribution graphical representation of the relative abundance of bacterial diversity from phylum to species can be visualized using Krona chart. UPGMA Clustering is a type of hierarchical clustering method using mean linkage and can be used to interpret the distance matrix. Statistical analysis to confirm differences in the abundance of individual taxonomy between the two groups, STAMP software was used. LEfSe was used for quantitative analysis of biomarkers in different groups. This method was designed to analyze data in which the number of species is much higher than the number of samples and to provide biological class explanations to establish statistical significance, biological consistency, and effect size estimation of predicted biomarkers. To identify differences in microbial communities between the two groups, ANOSIM and ADONIS were performed based on Bray–Curtis dissimilarity distance matrices.

### Statistical analysis

Sample size was calculated using the power analysis program G*Power (a-error ¼ 0.05, power ¼ 0.80). Animals were randomly divided into experimental groups and all experimental measurements were performed by two blinded investigators. Statistical analyses were performed using GraphPad Prism 9 (GraphPad Software Inc.). The normality of the data was analyzed prior to testing. If not mentioned otherwise, we used the t-Test or Mann–Whitney test to compare two conditions and (multiple) comparison tests in one/two-way ANOVA analyses or Kruskal–Wallis for more than two groups.

## Results

### LP and LB improved EAN

**Fig. 1:**
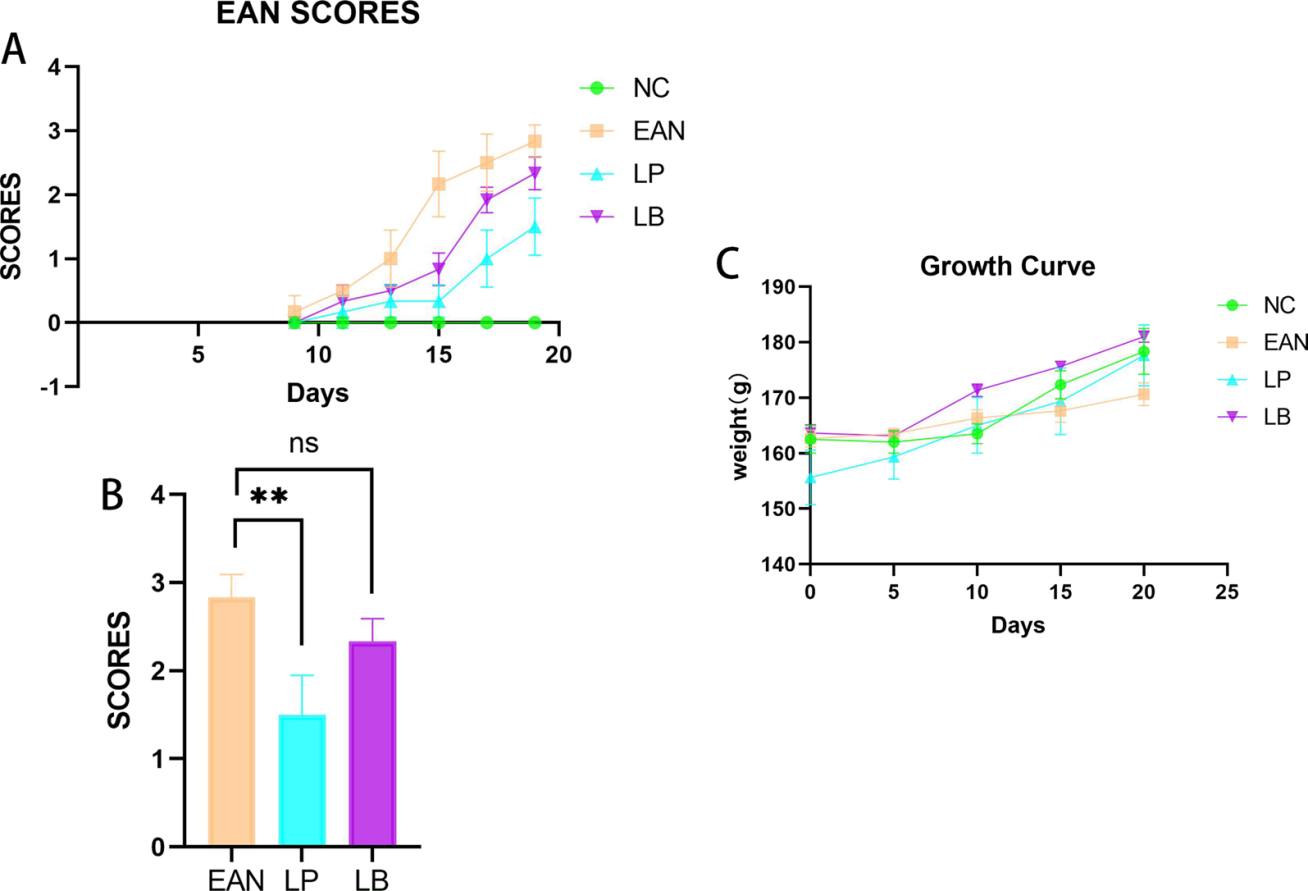
LP treatments improved EAN status (*n* = 6). **A**, **B** Show the scores of four groups. **C** Shows no statistically significant difference in weight change between the treatment groups and the model group during the disease progression stage. (*p < 0.1,**p < 0.01, *** *p* < 0.001,****p < 0.0001)

To investigate the therapeutic effect of *Lactobacillus* on EAN, animals in the EAN group and the treatment groups were administered 300 µg P0180-199 emulsified to a complete Freund adjuvant (10 mg/mL *M. tuberculosis* H37Ra) with bilateral plantar injection of 150 µL per rat on days 1 and 7 of immunization. The treatment group started gavage LP or LB (5 × 10^9^ CFU/mL) daily for one week before immunization, and the model group and the control group started gavage saline. Onsets were observed on day 9 postimmunization in the EAN group and on day 11 in the treatment groups, LB and LP treatments could delay the onset of the disease (Table 1). Disease progression was rapid, peaking at day 19 postimmunization, and the score was 1.5 ± 0.408 in the LP treatment group, 2.33 ± 0.235 in the LB treatment group, and 2.833 ± 0.235 in the EAN group on day 19 postimmunization. The scores in the LP treatment groups were significantly lower than in the EAN group (*p* < 0.05) (Fig. 1B). Weights were recorded between treatment and model groups during disease progression and there was no statistically significant difference in body weight (Fig. 1C). The area under the curve was calculated as 15.33 ± 1.221 in the EAN group, 5.167 ± 1.000 in the LP group, and 9.5 ± 0.646 in the LB group. Both LP and LB improved the disease process (*p* < 0.05), but LB only worked in the early stages and had little effect on the later stages of the disease (Fig. 1A).

**Table 1:** EAN cycle score table

Dates	NC (scores)	EAN (scores)	EAN + LP (scores)	EAN + LB (scores)
9 days 11 days 13 days 15 days 17 days 19 days	0 0 0 0 0 0	0.16 ± 0.235 0.50 ± 0 1.00 ± 0.408 2.17 ± 0.471 2.50 ± 0.408 2.83 ± 0.235	0 0.16 ± 0.235* 0.33 ± 0.235** 0.33 ± 0.235*** 1.00 ± 0.408** 1.50 ± 0.408**	0 0.23 ± 0.235* 0.5 ± 0* 0.83 ± 0.37** 1.83 ± 0.35 2.33 ± 0.235

*p < 0.1,**p < 0.01, *** *p* < 0.001

### LP and LB improved the ratio of CD4^+^T/CD8^+^T cells in peripheral blood

**Fig. 2:**
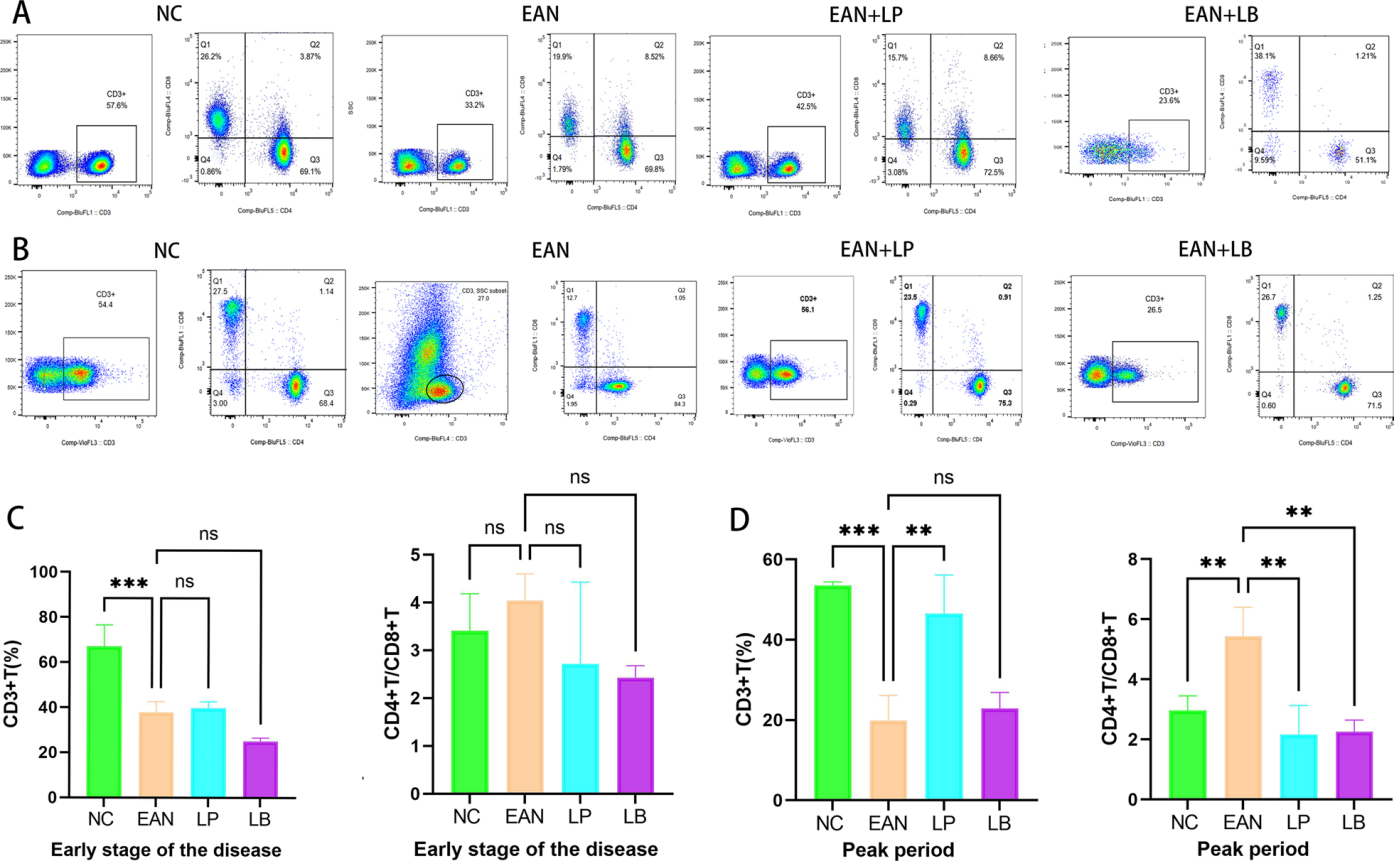
Flow cytometry analysis of CD3^+^T, CD4^+^T/CD8^+^T in peripheral blood mononuclear cells at onset and peak of pathogenesis (*n* = 6). **A** and **C** The result of CD3 ^+^ T and CD4^ +^ /CD8 ^+^ T cells in peripheral blood mononuclear cells in the initial stage of pathogenesis. **B** and **D** The result of CD3 ^+^ T and CD4 ^+^ /CD8 ^+^ T cells at the peak of pathogenesis in peripheral blood mononuclear cells. (*p < 0.1,**p < 0.01, *** *p* < 0.001,****p < 0.0001)

CD4 ^+^ T/CD8 ^+^ T ratio was a basic indicator of immune regulation and also played an important role in autoimmune disease [21]. EAN was an autoimmune disease dominated by CD4^+^T cells and macrophage infiltration [22]. Upregulation of the CD4^+^T was usually associated with EAN, so we used the CD4^+^T/CD8^+^T cell ratio to monitor immune balance in EAN [23–25]. At 11th (early onset) and 19th (peak) after immunization, tail vein blood was collected to detect CD3^+^CD4^+^T and CD3^+^CD8^+^T. In the early stages of onset, CD4^+^T was 69.1% and CD8^+^T was 26.2% in the NC group; CD4^+^T was 69.8% and CD8^+^T was 19.9% in the EAN model group; CD4^+^T was 72.5% and CD8^+^T was 15.7% in the LP treatment group; CD4^+^T was 51.1% and CD8^+^T was 38.1% in the LB treatment group (Fig. 2A). At the peak of incidence, CD4^+^T was 68.4% and CD8^+^T was 27.5% in the NC group; CD4^+^T was 84.3% and CD8^+^T was 12.7% in the EAN model group; CD4^+^T was 75.3% and CD8^+^T was 23.5% in the LP treatment group and in the LB treatment group, CD4^+^T was 71.5% and CD8^+^T was 26.7% (Fig. 2B). At 11th (early onset) after immunization, CD3^+^T in the EAN model group was lower than that of normal control (*p* < 0.05), but the CD4^+^T/CD8^+^T ratio was normal (Fig. 2C). This may be due to the fact that the disease was in the early stages of infection, the body was in a vulnerable state, and the immune balance was not yet out of balance. On day 19 after immunization (peak), in EAN group, CD3^+^T ratio, CD4^+^T/CD8^+^T was increased, LP and LB could improve the immune imbalance. The decrease in CD3^+^T cells may be related to the infiltration of a large number of T cells into peripheral nerve tissue.

### LP and LB improved the release of inflammatory factors in peripheral blood

**Fig. 3:**
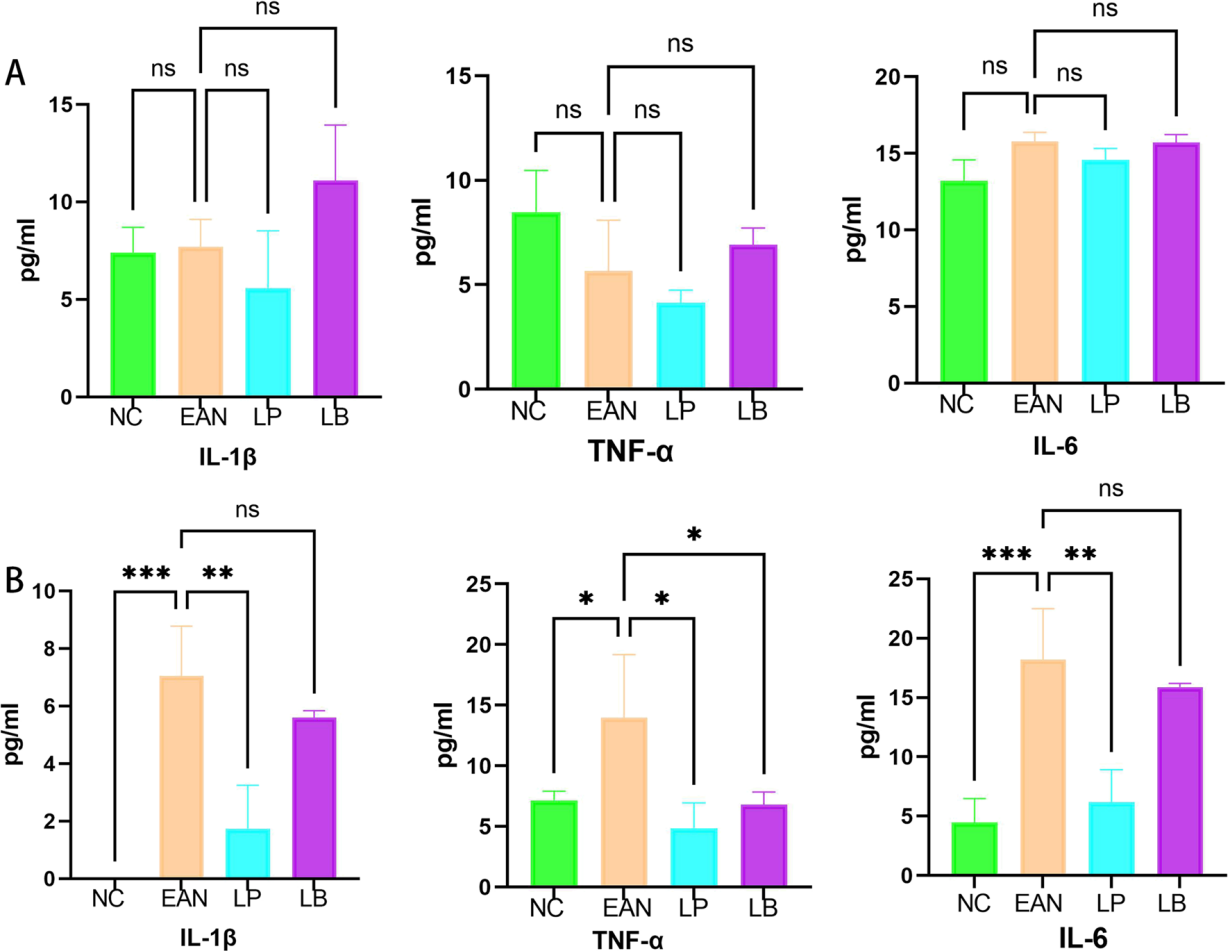
Effects of LP and LB on release of inflammatory factors (*n* = 6). **A** The levels of IL-1, IL-6, TNF-α in peripheral blood at the beginning of the disease (day 11 of immunization), and the LP treatment group was able to downregulate TNF-α and IL-6 compared with the model group, but there was no statistical significance (*p* > 0.1). **B** IL-1, IL-6, TNF-α levels measured by peripheral blood during the peak incidence period (day 19 of immunization). At peak, the levels of inflammatory factors were upregulated in the EAN model group, while levels of all inflammatory factors were downregulated in the LP treatment group, and expression of TNF-α was downregulated by LB. (*p < 0.1, **p < 0.01, ***p < 0.001, ****p < 0.0001)

Peripheral blood was collected from the tail vein at the beginning of the disease (11th day after immunization) and at the peak of the disease (19th day after immunization). Supernatant was taken after centrifugation to detect IL-1, IL-6 and TNF-α levels. In the early stages of pathogenesis, IL-1, IL-6 and TNF-α in peripheral blood did not change significantly (Fig. 3A). At the peak of the disease, the EAN model group significantly upregulated IL-1, IL-6, TNF-α, and the LP treatment group could downregulate IL-1, IL-6 and TNF-α, while LB had little effect on IL-1, IL-6 in peripheral blood (Fig. 3B).

### Improvement of pathological changes in sciatic nerve after treatment with LP or LB

**Fig. 4:**
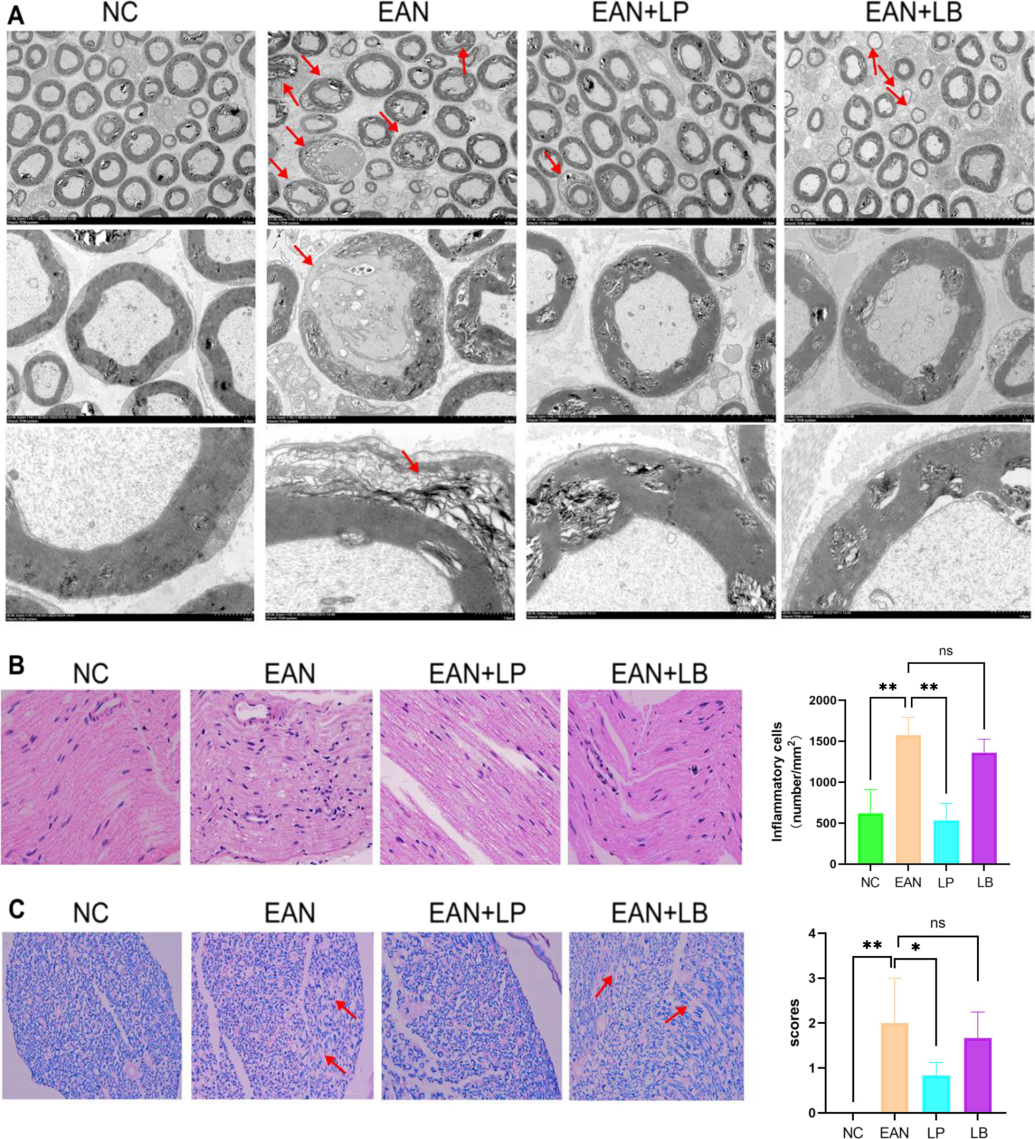
Pathological changes in sciatic nerve (*n* = 6). **A** Sciatic nerve tissue structures with different magnification under transmission electron microscopy (1000×, 2000×, 10,000×). **B** HE staining of the sciatic nerve. **C** The LFB staining of the sciatic nerve. (*p < 0.1,**p < 0.01, ***p < 0.001,****p < 0.0001)

At peak (day 19 after immunization), the rat sciatic nerve was taken, and HE staining and LFB staining were performed after section embedding to assess the degree of inflammatory cell infiltration and demyelination of the sciatic nerve, while pathological changes of the sciatic nerve were assessed by electron microscopy after sectioning (Fig. 4). The results showed that the axons of nerve fibers in the control group were completely arranged in order, the morphology of myelin sheath was regular, and the morphology of nucleus was normal (Fig. 4A). The nerve axons of the EAN model group were incomplete, the myelin sheath was shed, and the morphology was clearly irregular and loosely arranged. Compared with the EAN model group, the LP treatment group had a regular axon arrangement and improved myelin morphology. However, the sciatic nerves in the LB treatment group were loosely arranged, and the thickness of the myelin sheath was significantly thinner (Fig. 4A). The HE staining results showed that the inflammatory cell infiltration in EAN group was evident, and inflammatory cell infiltration was significantly improved in the LP treatment group. LB had no obvious effect on inflammatory cell infiltration. In the EAN model group, the number of inflammatory cell infiltrates per mm^2^ was 1573.333 ± 179.1337, the number of inflammatory cell infiltration in the LP treatment group was 533.33 ± 169.9673, and the number of inflammatory cell infiltration in the LB treatment group was 1360 ± 133.6663 (Fig. 4B). Figure 4C is the LFB staining of the sciatic nerve, and the results showed that the myelin sheath in the control group was intact and neatly arranged, while it was loosely arranged and had a large area of myelin shedding in the EAN model group, and the LP treatment group improved the myelin shedding situation. There was no significant improvement in EAN demyelination in the LB treatment group. There was a statistical analysis of the neural demyelinating staining score, with 1 being mild demyelinating, 2 moderately demyelinating, and 3 severely demyelinating (Fig. 4C).

### Effects of LB and LP on the intestine

**Fig. 5:**
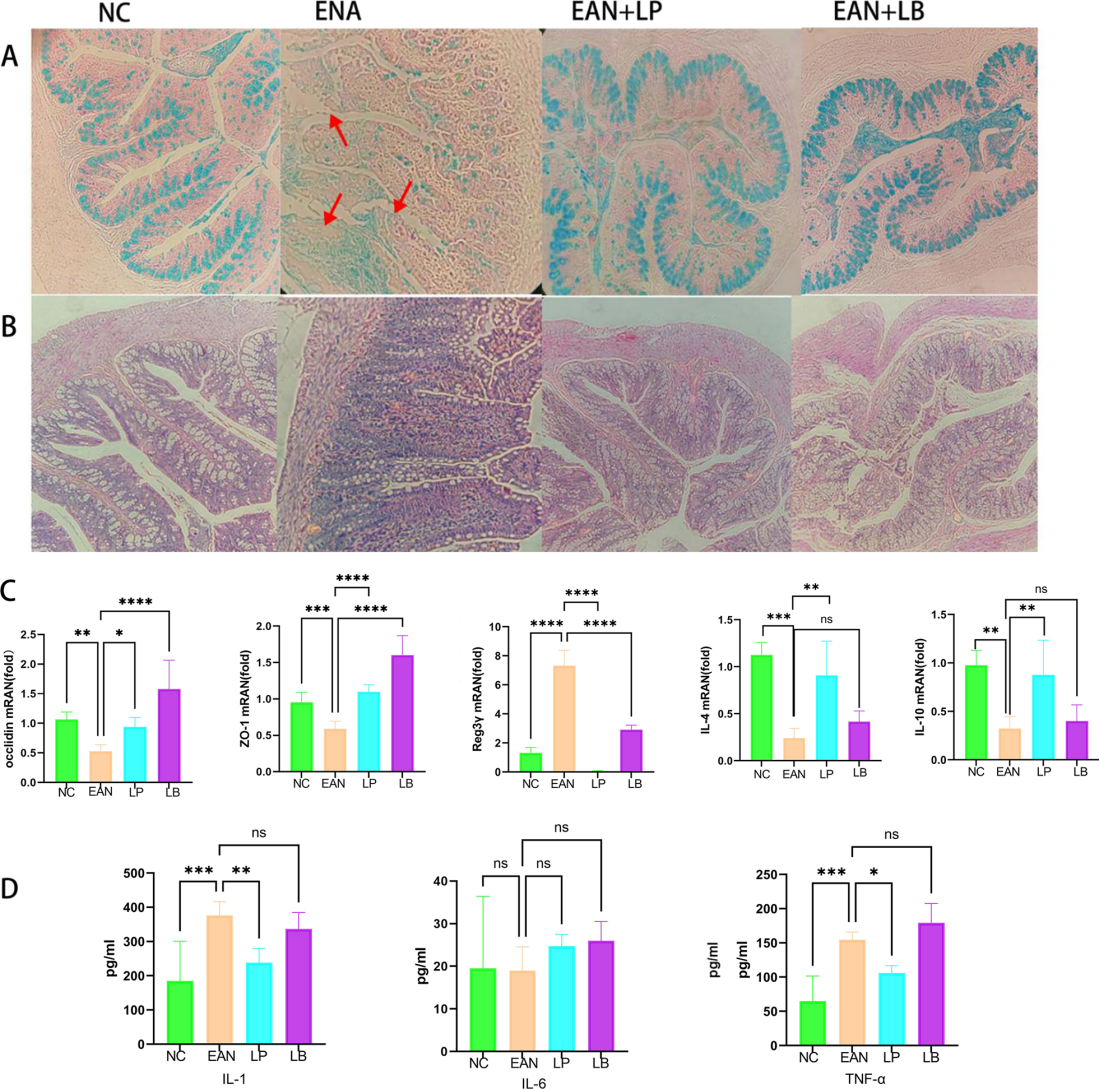
Pathological changes in intestinal mucosa and occludin, ZO-1, reg3γ,IL-4, IL-10 mRNA levels and IL-1, IL-6, TNF-α inflammatory factor levels (*n* = 6). **A** HE staining. **B** Alixinlan staining. **C** Occludin, ZO-1 and Reg3γ, IL-4, IL-10 mRNA levels. **D** The statistical results of intestinal IL-1β, IL-6 and TNF-α. (*p < 0.1, **p < 0.01, *** *p* < 0.001, ****p < 0.0001)

Colonic specimens were taken at the peak of the disease for HE staining and Alixinlan staining to observe inflammatory infiltration and mucosal integrity. The results (Fig. 5A, B) showed that intestinal mucosa was destroyed, structural disorder and inflammatory cell infiltration were evident in the EAN model group, while intestinal inflammation in the LB or LP treatment group was significantly improved, and intestinal mucosal structure was regular. At the same time, the expression of occludin, ZO-1, reg3γ, IL-4 and IL-10 genes was detected at mRNA level (Fig. 5C), and IL-1, IL-6 and TNF-α gene expression levels were detected by ELISA (Fig. 5D). The results showed that occludin, ZO-1, IL-4 and IL-10 gene expression levels were significantly downregulated in the EAN model group, while the LP or LB treatment group improved occludin and ZO-1 to be close to NC normal control group. IL-4 and IL-10 could be upregulated by LP but not LB. Reg3γ in the EAN model group was significantly upregulated, while reg3γ could be downregulated in the LB or LP treatment group. The EAN model group upregulated IL-1β and TNF-α, while the LP-treated group improved this upregulation (*p* < 0.05). Results indicated that LP had a pronounced anti-inflammatory effect and the ability to repair the intestinal barrier.

### Fecal differential metabolite analysis

**Fig. 6:**
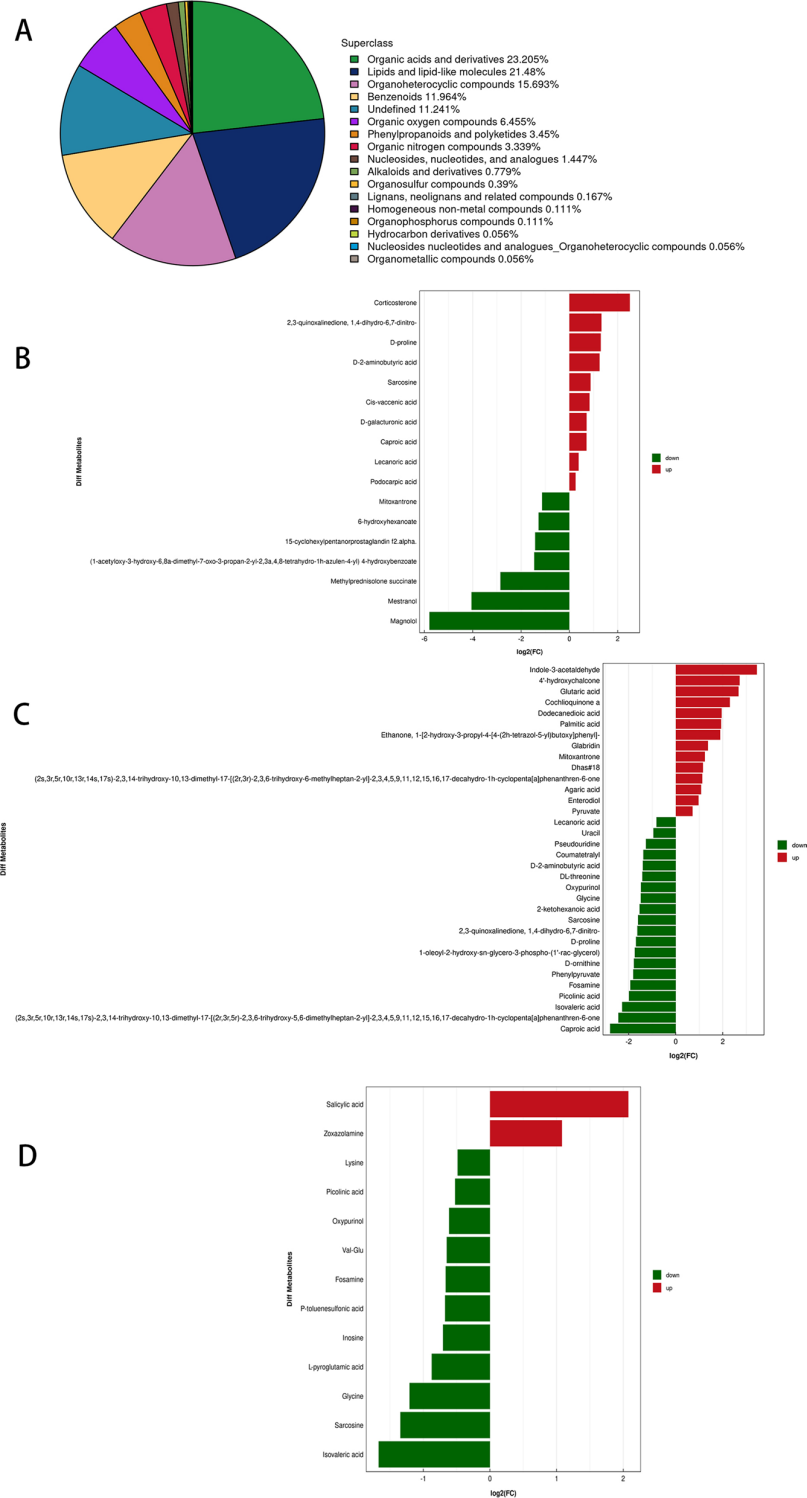
Differential metabolites and their classification. **A** The 1797 metabolites identified by collecting rat feces for metabolomic determination, which were classified and counted according to their chemical classification (Chemical Taxonomy) attribution information. **B** 17 differential metabolites in the EAN model group and NC group. **C** 34 differential metabolites in the LP treatment group and ENA model group. **D** 13 differential metabolites in the LB treatment group and ENA model group with strict OPLS-DA VIP > 1 and *p* < 0.05 as significant differential metabolites

Rat feces were collected for metabolomic determination, and 1797 metabolites were identified, which were classified as lipids (23.205%), lipid-like molecules (21.48%), organic heterocyclic compounds (15.693%), and benzene compounds (11.964%) according to their chemical taxonomy attribution information, organic acids and derivatives (Fig. 6A). Among NC group and EAN group, 2,3-quinoxalinedione, 1,4-dihydro-6,7-dinitro-, Cis-vaccenic acid, podocarpic acid, d-galacturonic acid, sarcosine, corticosterone, d-2-aminobutyric acid, d-proline, lecanoric acid were upregulated. Magnolol, mitoxantrone, methylprednisolone succinate, mestranol, 4-hydroxybenzoate, 6-hydroxyhexanoate, cyclohexylpentanorprostaglandin were downregulated (Fig. 6B). There were 34 metabolic differences between the LP treatment group and the EAN model group, among which the upregulated metabolites were indole-3-acetaldehyde, 4′-hydroxychalcone, glutaric acid, cochlioquinone acid, dodecanedioic acid, palmitic acid, ethanone, glabridin, mitoxantrone, et al. Downregulated are caproic acid, isovaleric acid, picolinic acid, fosamine, phenylpyruvate, d-ornithine, d-proline, 2,3-quinoxalinedione, 1,4-dihydro-6,7-dinitro-, sarcosine, 2-ketohexanoic acid, glycine, oxypurinol, dl-threonine, d-2-aminobutyric acid, coumatetralyl, pseudouridine, Lecanoric ACID, etc. 2,3-quinoxalinedione, d-proline, lecanoric acid, d-2-aminobutyric acid, sarcosine, and caproic acid that were upregulated in the EAN model group were all differentially downregulated after LP treatment (Fig. 6C). Mitoxantrone, which was downregulated in the EAN model group, was differentially upregulated in the LP-treated group. Indole-3-acetaldehyde, which was the most significant upregulation in the LP treatment group, was an NF-KB inhibitor, which inhibited the inflammatory pathway of NF-KB after upregulation. magnolol, was also an NF-KB inhibitor. Results suggested the anti-inflammatory effects of LP on the disease. Thirteen metabolites were altered in the LB treatment group, upregulated as salicylic acid, zoxazolamine, downregulated as lysine, picolinic acid, oxypurinol, val-glu, fosamine, p-toluenesulfonic acid, inosine, l-pyroglutamic acid, glycine, sarcosine, isovaleric acid. Fosamine was upregulated in EAN disease and downregulated in the LB treatment group (Fig. 6D). There were almost no metabolites associated with inflammation, and we hypothesized that LB had little effect on the development of inflammation in the disease. Zoxazolamine was a muscle relaxant, LB's remission effect on early stages of the disease may be related to zoxazolamine upregulation.

### KEGG differential metabolic pathway analysis

**Fig. 7:**
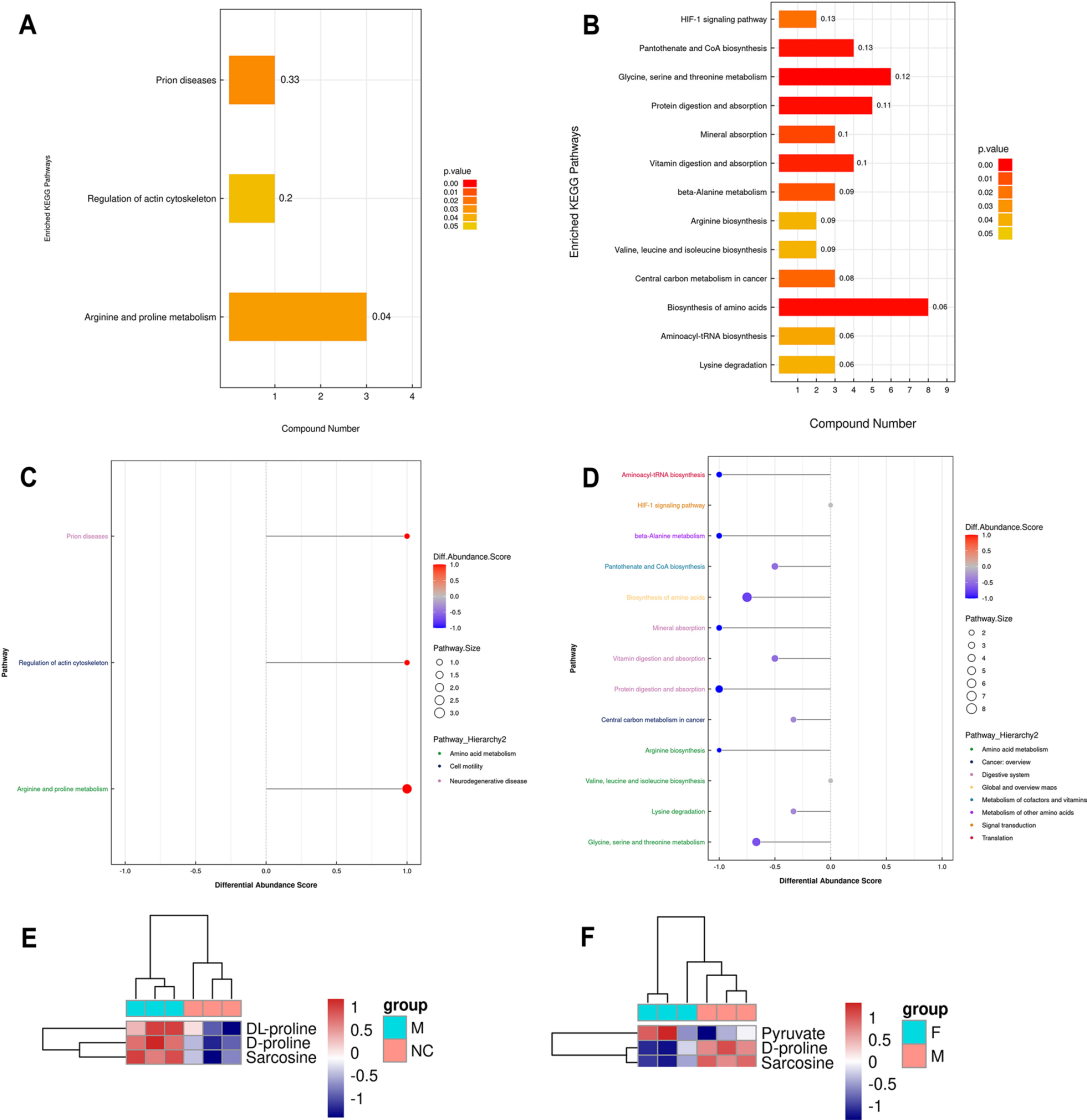
KEGG differential metabolic pathway analysis. **A**, **B**, Histograms of KEGG enrichment pathways, in which the vertical axis represented each KEGG metabolic pathway, and the horizontal axis represented the number of differentially expressed metabolites contained in each KEGG metabolic pathway. **C**, **D**, The Differential Abundance Score, which was proportional to the color of the dot and the DA score value. **E**, **F**, A clustered heat map of differential metabolites of the KEGG pathway, and the color block at different locations represented the relative expression of metabolites

KEGG (Kyoto Encyclopedia of Genes and Genomes, http://www.kegg.jp/) is one of the most commonly used databases of gene pathways. KEGG database includes pathway information, genetic information processing, environmental information processing, cellular processes, biological systems, human diseases, and drug development. The results of metabolic pathway enrichment analysis were presented in a histogram, and the EAN model group was enriched in three metabolic pathways. These included Arginine and proline metabolism, Regulation of actin cytoskeleton, and prion diseases (Fig. 7A). Differential metabolites in the LP group were enriched for biosynthesis of amino acids; glycine, serine and threonine metabolism; protein digestion and absorption; pantothenate and CoA biosynthesis; vitamin digestion and absorption; beta-alanine metabolism; mineral absorption; lysine degradation; aminoacyl-tRNA biosynthesis; central carbon metabolism in cancer; valine, leucine and isoleucine biosynthesis; arginine biosynthesis; HIF-1 signaling pathway (Fig. 7B). The Differential Abundance Score of the enriched pathways showed that the metabolic pathways of the differential metabolites in the EAN model group were significantly upregulated, while the metabolic pathways of the differential metabolites in the LP treatment group were significantly downregulated (Fig. 7C, D). In order to facilitate the observation of the expression of each differential metabolite annotated in the KEGG metabolic pathway, the KEGG metabolic pathway with a number of differential metabolites greater than 2 was selected in this study, and co-metabolic differences containing LP and EAN were screened, and the differential metabolites in the KEGG metabolic pathway were displayed as heat maps, and the results showed that the differential metabolites dl-proline, d-proline, sarcosine significantly upregulated in arginine and proline metabolism. Upregulation of the differential metabolite pyruvate in the LP-treated group and downregulation of d-proline and sarcosine enriched the same pathway in arginine and proline metabolism, which was downregulated in the LP-treated group compared to the EAN model group (Fig. 7E, F).

### Analysis of microbial community structure and differences

**Fig. 8:**
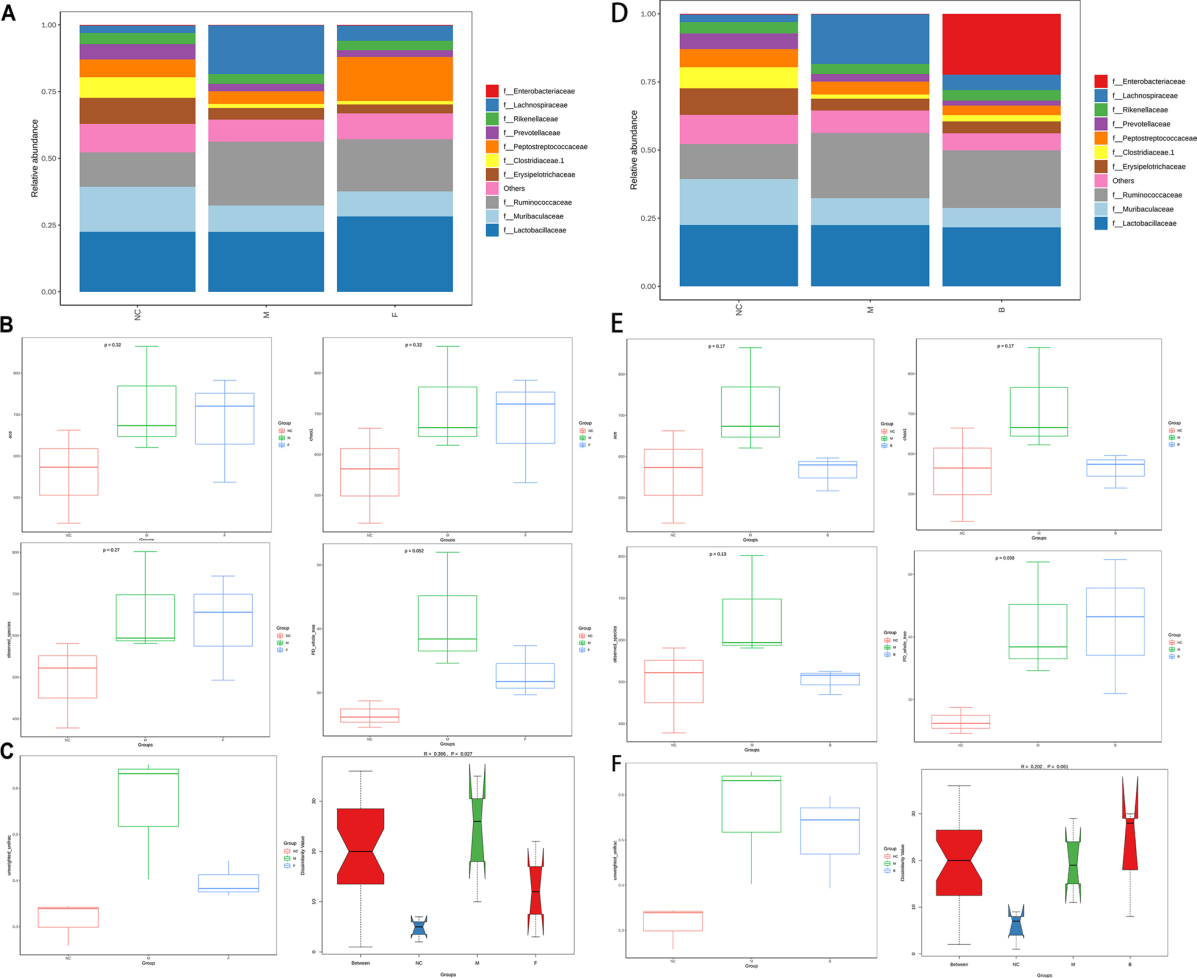
Analysis of species and differential functions in microbial community. **A**–**C** For LP treatment group with analysis of EAN disease; **D**–**F** for LB treatment group with EAN disease analysis. **A** The top ten species at the family level. **B** Alpha diversity analysis (CHAO 1, ACE index, observed OUT, PD whole tree). **C** The analysis of β diversity. **D** The top ten species at the family level. **E** Alpha diversity analysis (CHAO 1, ACE index, observed OUT, PD whole tree). **F** β diversity analysis

The Community Structure Component Map showed the community structure of each group at different classification levels. *Lactobacillaceae* accounted for 22.5%, *Ruminococcaceae* 12.8%, *Muribaculaceae* 16.8%, and *Lachnospiraceae* 2.7% in the normal control group. In the EAN model group, *Lactobacillaceae* accounted for 22.4%, *Ruminococcaceae* 23.8%, *Muribaculaceae* 9.8%, and *Lachnospiraceae* 18.2%. In the LP treatment group, *Lactobacillaceae* accounted for 28.2%, *Ruminococcaceae* 19.4%, *Muribaculaceae* 9.3%, and *Lachnospiraceae* 5.6% (Fig. 8A). In the LB treatment group, *Lactobacillaceae* accounted for 21.7%, *Ruminococcaceae* 21.1%, *Muribaculaceae* 7.1%, and *Lachnospiraceae* 5.6% (Fig. 8D). The EAN model group upregulated Rumino*coccaceae*, *Lachnospiraceae*, While the LP and LB treatment group downregulated *Ruminococcaceae*, *Lachnospiraceae*. The EAN model group downgraded *Muribaculaceae*, and LB drops even more. Alpha Diversity was used to analyze the diversity of microbial communities within the sample (Within-Community), and there was no statistically significant difference in the results indicators CHAO1, ACE index, Observed OUT, PD whole tree (*p* > 0.05) (Fig. 8B). Further Anosim analysis was performed based on unweighted UnifracBeta distance to analyze the β diversity. Further Anosim analysis was a nonparametric test to test whether the differences between groups were significantly greater than the differences between groups, and the results showed that the group community structure had significant differences (Fig. 8C). Figure 8E, F shows the effect of intestinal flora in LB treatment group, and it could be seen that LB had almost little effect on Alpha diversity (CHAO1, ACE index, Observed OUT, PD whole tree) and β diversity (*p* > 0.05).

## Discussion

The purpose of this study was to investigate the effect of oral administration of LP and LB on EAN in Lewis rats and to develop a new therapeutic method. Our results showed that after a long-term diet concept (early prevention), receiving LP and LB seven days before immunization and continuing after immunization with usual food dosages leaded to amelioration of the disease course. LP reduced inflammation and demyelination in histology. We speculated that this was related to the reduction of IL-1, IL-6, TNF-α inflammatory factor levels by LP and the dynamic regulation of CD4^+^/CD8^+^T cell balance. It was consistent with existing studies showing that LP modulated immune response and anti-inflammatory properties [19, 26–29]. Treatment with LP induced a significant improvement in inflammatory status by reducing serum lipopolysaccharide (LPS), free fatty acids (FFA), TNF-α, IL-6, and IL-8 levels, and increasing IL-10 levels [30]. Also, studies have reported that LP improves neuroinflammation [31], and prevents the development of allergies by modulating the host immune response [32, 33].LB had a relatively small role in anti-inflammatory, mainly manifested in the downregulation of TNF-α. In EAN, main upregulated cytokine was TNF-α, TNF-α induced matrix metalloproteinase (MMP-9) promoted macrophage recruitment into damaged peripheral nerves [34]. Our study proved that LB had a regulatory effect on CD4^+^T, and speculated that the level of TNF-α was downregulated mainly by regulating CD4^+^ T cells. The downregulation of TNF-α affected the expression of IL-1 and IL-6. Meanwhile, LP upregulated CD3^+^T cells in peripheral blood of EAN, while LB could not. The decrease of CD3^+^T in peripheral blood was related to the infiltration of sciatic nerve T cells and inflammatory cells, LP improved the inflammatory cell infiltration and demyelinating of EAN, the content of CD3^+^T in tissues was reduced, and the content of peripheral blood was increased. LB had less effect on inflammation and demyelination.

Potential mechanisms by which disturbance of gut flora leaded to autoimmune diseases (AID) [35]. To avoid overactivation of the immune system, the integrity of the intestinal barrier was necessary to maintain the balance between microbial components and host immunity [36]. However, the intestinal wall of patients with AID was often accompanied by damage and leakage [37], which was consistent with our findings. In EAN, intestinal mucosa was disrupted, inflammatory factors IL-1, IL-6, TNF-α were upregulated, occludin and ZO-1 are downregulated, but confusingly Reg3γ levels are significantly upregulated. It has been reported that tight junction proteins ZO-1 and occludin may become indicators to assess the degree of inflammation in the ulcerated intestine and predict mucosal healing, the lower the ZO-1 and occludin expression, the more serious the intestinal mucosal damage [38–40]. LP improved intestinal mucosa damage and permeability by upregulating ZO-1 and occludin protein and downregulating inflammatory factors, and LB improved intestinal pathological injury by upregulating ZO-1 and occludin protein. In addition, Reg3γ was an antimicrobial protein associated with inflammation, and we speculated that upregulation of Reg3γ expression in EAN was associated with increased intestinal inflammation [41]. Impaired intestinal barrier increases the transfer of gut microbes and their components, leading to abnormal contact between gut microbes and the host immune system and triggering autoimmunity through various mechanisms.

Many studies have found that as research on gut microbiota advances, the gut microbiota in AID patients with AID has also been discovered to be significantly different from healthy people [42, 43]. Some species that colonize the human gut, such as *Prevotella copri*, *Ruminococcus gnavus* and *L. salivarius*, have also been associated with the onset of AID [44]. In our study, *Ruminococcaceae* and *Lachnospiraceae* were upregulated in the EAN model group. LP and LB downregulated *Ruminococcaceae* and *Lachnospiraceae*, which may be related to LP and LB's ability to maintain immune balance. Specific strains of gut bacteria could drive body-specific autoantibody production and joint-centric antibody deposition and immune activation, stimulating Th17 cell activation, such as identifying cross-activity between RA-relevant autoantigens and bacterial taxa in the closely related families *Lachnospiraceae* and *Ruminococcaceae* [45]. But, *Muribaculaceae* was regulated by LP not LB. Studies have reported that *Muribaculaceae*, the main flora that produces short-chain fatty acids, is positively correlated with the recovery of neuroinflammation [46–48]. The results suggested the different mechanisms of LP and LB action on EAN. Both LP and LB changed the organization of the community structure, the LP community structure was closer to the normal community, which improved the EAN dysbacteriosis.

Microbial derivatives mediated dysregulation of immune response. The results of this study showed that D-Proline and Sarcosine, the core differential metabolite, were significantly upregulated in the EAN model group and downregulated significantly in the LP treatment group, which was enriched in a metabolic pathway, arginine and proline metabolism. However, these metabolites were not downregulated in the LB treatment group, which may be related to LB's smaller effect on the disease. There is growing evidence that abnormal immune cell metabolism in AID promotes cellular and molecular processes of inflammation [49], intracellular arginine concentration directly affects the metabolic adaptability and viability of T cells, which are closely related to EAN [50]. In EAN, the decisive role of T-lymphocytes in initiating immune-mediated nerve damage has been firmly established by adaptive transfer experiments. Macrophages but not Schwann cells express major histocompatibility complex class II gene products in situ and may therefore function as antigen presenters. Macrophages are critical in the amplification and effector phase and damage the myelin sheath by phagocytic attack and release of inflammatory mediators such as toxic oxygen radicals, arachidonic acid metabolites, complement, or hydrolases [51]. Macrophage polarization is accompanied by dramatic changes in L-arginine metabolism [52]. The third metabolic fate of l-arginine is creatine generation, which acts as a key source of cellular energy reserve, yet little is known about the role of creatine in the immune system. Here, genetic, genomic, metabolic, and immunological analyses revealed that creatine reprogrammed macrophage polarization by inhibiting M (IFN-γ) yet promoting M (IL-4) effector functions [53]. In summary, arginine and proline metabolism are closely related to T cell differentiation and macrophage polarization, which is consistent with our results.

## Conclusion

By establishing an EAN model in Lewis rats, LP affected intestinal microbial structure (*Ruminococcaceae, Lachnospiraceae* and *Muribaculaceae*) regulated inflammatory response and immune response. In addition, cellular arginine and proline metabolism was regulated by altering gut microbial metabolites (D-proline, sarcosine), thereby affecting T cell activation and macrophage polarization. Meanwhile upregulating indole-3-acetaldehyde, magnolol, inhibited NF-KB inflammatory pathway and improved disease progression. LB may affect the structure of intestinal microorganisms and repair intestinal mucosa, but the effect on disease was not significant. This study provided new strategies for the treatment of the disease.

## Data Availability

The data of this study are available from the corresponding author, upon reasonable request.
